# EGDMA- and TRIM-Based Microparticles Imprinted with 5-Fluorouracil for Prolonged Drug Delivery

**DOI:** 10.3390/polym14051027

**Published:** 2022-03-04

**Authors:** Michał Cegłowski, Joanna Kurczewska, Aleksandra Lusina, Tomasz Nazim, Piotr Ruszkowski

**Affiliations:** 1 Faculty of Chemistry, Adam Mickiewicz University, 61-614 Poznan, Poland; asiaw@amu.edu.pl (J.K.); aleksandra.lusina@amu.edu.pl (A.L.); tomasz.nazim@amu.edu.pl (T.N.); 2 Department of Pharmacology, Poznan University of Medical Sciences, 61-614 Poznan, Poland; pruszkowski@gmail.com

**Keywords:** molecularly imprinted polymers, microspheres, drug delivery, 5-fluorouracil

## Abstract

Imprinted materials possess designed cavities capable of forming selective interactions with molecules used in the imprinting process. In this work, we report the synthesis of 5-fluorouracil (5-FU)-imprinted microparticles and their application in prolonged drug delivery. The materials were synthesized using either ethylene glycol dimethacrylate (EGDMA) or trimethylolpropane trimethacrylate (TRIM) cross-linkers. For both types of polymers, methacrylic acid was used as a functional monomer, whereas 2-hydroxyethyl methacrylate was applied to increase the final materials’ hydrophilicity. Adsorption isotherms and adsorption kinetics were investigated to characterize the interactions that occur between the materials and 5-FU. The microparticles synthesized using the TRIM cross-linker showed higher adsorption properties towards 5-FU than those with EGDMA. The release kinetics was highly dependent upon the cross-linker and pH of the release medium. The highest cumulative release was obtained for TRIM-based microparticles at pH 7.4. The IC_50_ values proved that 5-FU-loaded TRIM-based microparticles possess cytotoxic activity against HeLa cell lines similar to pure 5-FU, whereas their toxicity towards normal HDF cell lines was ca. three times lower than for 5-FU.

## 1. Introduction

Drug delivery systems (DDS) are sophisticated technologies that allow targeted delivery of particular pharmaceutical or controlled therapeutic agents’ release. DDS application is essential for therapeutic agents limited due to their low solubility, drug administration issues, or very fast metabolism. Moreover, the application of DDS allows to optimize drug efficiency and improve administration, while lowering the possibility of causing adverse side effects. As a result, the development of new DDS occurs in parallel with creating new therapeutic agents. Whenever a new drug has a limited therapeutic effect in a free form, the combination of drugs and DDS may overcome this problem. The design, synthesis, and production of new substances and materials that can be applied to prepare new DDS have become a major topic of many research groups. Their goal is to develop a DDS which would allow drug administration to a specific site with a known quantity and for a precise amount of time. It is, of course, necessary to produce DDS that, as well as their metabolites, show no toxicity and are easily removed from the human body [1,2,3].

Various materials have been used to generate DDS. The most frequently used are lipids [4,5], chitosan [6,7], silica [8,9,10], halloysite [11,12], functional polymers [13,14,15,16,17,18,19], and dendrimers [20,21]. Among the described materials, functional polymers are frequently used due to their high synthetic versatility, a broad range of final properties, and various possible applications [22,23]. Sophisticated polymeric DDS should guarantee drug delivery in a predesigned manner. Functional polymers can be designed to fulfill the most sophisticated criteria of DDS. They can be synthesized to become triggered-release DDS or release a drug following a particular kinetic profile. As a result, it is possible to obtain passive or active targeting, which means that the whole system is responsible for the therapeutic benefit. Molecularly imprinted polymers (MIPs) represent a group of functional polymers that can be easily tuned to possess a defined affinity to a drug molecule, and thus they often find application in producing new DDS [24,25,26].

MIPs are synthesized by copolymerizing a cross-linker mixed with a complex formed by functional monomers and template molecules. The cross-linker initiates the formation of the bulk polymer structure and entrapment of the template molecules inside, whereas functional monomers form stable, non-covalent interactions with template molecules. Finally, it results in the synthesis of MIPs selective towards the template used, which can be reversibly bound by cavities formed during the reaction. As a result, they have found many applications in fields such as solid-phase extraction (SPE) [27,28,29], development of sensors [30,31,32], and drug delivery [33,34].

Many researchers find the application of MIPs in drug delivery particularly interesting due to the possibility of producing materials that alter their interaction strength with drug molecules influenced by a specific change in the environment. MIPs possess an enhanced affinity to the drug template, which increases the residence time of the drug. Moreover, MIPs’ properties allow for reaching high drug loading, and the fact that the drug is entrapped within the polymeric network results in its higher stability and durability against harsh conditions. MIPs can be synthesized as smart materials that can be effectively used to produce DDS, considering that they can be programmed to release therapeutic agents as a response to defined stimuli [35]. MIPs can also be synthesized to develop materials that release molecules entrapped inside cavities with a particular kinetic profile [36]. This can be achieved by selecting the appropriate type and amount of functional monomers present in the MIPs’ structure. Our group recently published a detailed investigation about using MIPs for prolonged drug delivery of doxorubicin [37] and paclitaxel [38].

MIPs are synthesized as monoliths that are ground to obtain particles of desired dimensions for numerous applications. Although this process is frequently used in analytical applications such as SPE, the development of DDS requires more rigorous control over polymer particles’ size. New synthetic methods that allow obtaining MIPs as uniform nanoparticles or microparticles have been developed to solve this problem. As synthesized materials possess a significantly increased surface area, more cavities are closer to the surface, making them easily accessible. The additional benefit of small particle sizes is their ability to form stable dispersions in various solvents, which increases their attractiveness as potential materials to be used as DDS [39,40,41].

In this work, we report the synthesis and characterization of MIPs microparticles imprinted with 5-fluorouracil (5-FU). 5-FU is a cytotoxic drug with a broad activity spectrum against numerous tumors. It undergoes fast metabolism in the human body, and thus a therapy consisting of high doses is required, which can cause severe toxic effects in many patients [42]. As a result, new DDS for 5-FU are being developed [43]. The advantage of using microparticles for drug delivery is that they do not traverse into the interstitium over the size of 100 nm transported by the lymph, and thus they only act locally in a place of administration [44]. Methacrylate-based particulate carriers are widely applied for biomedical applications to obtain drug delivery systems. The therapeutic agent’s release from their structure typically occurs in a biphasic way with an incomplete drug release. The release mechanism can be described using both Fickian and non-Fickian kinetic models. The improvement of drug release can be achieved by increasing polymer hydrophilicity by synthesizing functional microspheres with additional functional groups or formulating composites with hydrophilic polymers [45]. The microparticles were synthesized by using precipitation polymerization. Methacrylic acid (MAA) was used as a functional monomer, whereas 2-hydroxyethyl methacrylate (HEMA) was used as a hydrophilic monomer to improve the water compatibility of the final materials. The microparticles were prepared using two cross-linkers, particularly ethylene glycol dimethacrylate (EGDMA) and trimethylolpropane trimethacrylate (TRIM), to examine the influence of the polymer matrix structure on the final properties of microparticles. The interactions of MIPs with 5-FU were investigated by examining their adsorption properties and release profiles of 5-FU. Finally, the in vitro activity of 5-FU-loaded MIPs against HeLa, U87 MG, A-549, KB, and MCF-7 cancer cell lines was investigated. The results were compared with the measurements performed against human dermal fibroblasts (HDF) normal cell lines. To the best of our knowledge, this is the first research describing the synthesis of 5-FU-imprinted hydrophilic microparticles and presenting their in vitro activity against cancer and normal cell lines.

## 2. Materials and Methods

### 2.1. Materials and Chemicals

Ethylene glycol dimethacrylate, methacrylic acid, trimethylolpropane trimethacrylate, 2-hydroxyethyl methacrylate, 5-fluorouracil, 2,2′-azobisisobutyronitrile solution (AIBN; 0.2 M in toluene), and all solvents (HPLC grade) were obtained from Sigma-Aldrich (St. Louis, MO, USA).

### 2.2. Instruments

The FTIR measurements were performed using a IFS 66/s spectrometer (Bruker, Billerica, MA, USA). To obtain spectra, 1.5 mg of each material was mixed with ca. 200 mg of KBr, and the resulting powder was converted into tablets used in FTIR measurements. Thermogravimetric (TG) analysis was performed using a Setsys 1200 (Setaram, Caluire, France) apparatus. The analysis was performed in an air stream (50 mL min^−1^) at a heating rate of 10 °C min^−1^. Scanning electron microscopy (SEM) images were recorded using a Scanning Electron Microscope SU3500 (Hitachi, Tokyo, Japan). UV-Vis absorption spectra were obtained using a 8453 (Agilent, Santa Clara, CA, USA) spectrophotometer. The solutions’ pH values were controlled with an CP-505 pH meter (Elmetron, Zabrze, Poland).

### 2.3. Synthesis of Microparticles

MIPs created using EGDMA cross-linker were synthesized as follows. The pre-polymerization mixture consisting of 5-FU (0.9 mmol), MAA (1.8 mmol), HEMA (0.9 mmol), and methanol (90 mL) was prepared in a glass pressure tube. The mixture was degassed for 30 min using an ultrasound bath and purging with inert gas (nitrogen). Subsequently, EGDMA (8 mmol) and AIBN solutions (1 mL) were added, and the tube was degassed for an additional 10 min. Afterward, the tube was sealed and placed at 60 °C for 18 h. As-synthesized microparticles were filtered off and dried under a vacuum. The resulting drug-loaded MIPs were placed in a dialysis tubing to remove template molecules and were dialyzed against acidified methanol (9:1 methanol/acetic acid). This process was continued until 5-FU was no longer detected in a dialysis solution. The drug-unloaded MIPs cross-linked with EGDMA (denoted as MIP_EGDMA_) were finally dried under a vacuum.

MIPs created using the TRIM cross-linker were prepared by the same setup and procedure as EGDMA-based MIPs, but with different amounts of reagents. The polymerization mixture consisted of 5-FU (2 mmol), MAA (4 mmol), HEMA (2 mmol), and methanol (80 mL). After degassing and sonication, TRIM (4 mmol) and AIBN solutions (1.4 mL) were added, and the tube was degassed for an additional 10 min. The remaining procedure is identical to EGDMA-based MIPs. Finally, the drug-unloaded MIPs cross-linked with TRIM were denoted as MIP_TRIM_.

The corresponding non-imprinted polymers (NIPs) were synthesized using analogous procedures but without adding template molecules. The obtained microparticles were denoted as NIP_EGDMA_ and NIP_TRIM_. The structures of all used monomers and templates are presented in Figure S1.

### 2.4. Adsorption Studies

Adsorption isotherms were established using batch experiments, in which 10 mL of methanolic 5-FU solution at concentrations ranging from 0.39 to 50 mg L^−1^ was added to 10 mg of MIPs or NIPs. The obtained mixtures were equilibrated for 24 h at ambient conditions (25 °C). UV-vis absorption spectra were taken prior to and after adsorption solutions to establish the concentration of 5-FU. The amount of adsorbed 5-FU (*q_eq_*, mg g^−1^) was calculated as follows:

(1) $$q_{eq}=\frac{\left(C_{0}-C_{eq}\right)V}{m}$$

where *C*_0_ is the initial concentration of 5-FU solution (mg mL^−1^), *C_eq_* is the equilibrium concentration of 5-FU solution (mg mL^−1^), *m* is the polymer (MIPs/NIPs) mass (*g*), and *V* is the volume of 5-FU solution (mL). The experiments were repeated three times, and mean values were used for calculation.

The adsorption kinetics studies were conducted when 50 mg of either MIPs or NIPs were placed in 50 mL of 5-FU solution in methanol with an initial concentration of 10 mg L^−1^. The solution was stirred at ambient conditions, and the concentration of 5-FU was measured at defined time intervals by UV-vis absorption spectra. The *q_t_* value (mg g^−1^), which represents the amount of adsorbed 5-FU, was calculated as follows:

(2) $$q_{t}=\frac{\left(C_{0}-C_{t}\right)V}{m}$$

where *C_t_* is the concentration of 5-FU after time *t* (h).

### 2.5. Release Experiments

The dissolution method was used to conduct the in vitro release studies. The experiments were conducted by dispersing 5-FU-loaded MIPs (20 mg) in buffer solutions (10 mL) at pH 2.2, 5.0, and 7.4. The solutions were stirred continuously for seven days at 37 °C, and at specified time intervals, the samples were collected, centrifuged, and their UV-vis absorption spectra were recorded. The obtained data allowed to calculate the amount of drug releases from studied MIPs. The experiments were repeated three times, and the obtained mean values were used for subsequent calculations.

The results obtained for the 5-FU release were fitted using various models that characterize the mechanism of the release process. The following models were applied: zero-order (Equation (3)), first-order (Equation (4)), simplified Higuchi (Equation (5)), Hixson–Crowell (Equation (6)), and Korsmeyer–Peppas (Equation (7)). The following equations mathematically represent these models:

(3) $$F_{t}=k_{0}t$$

(4) $$F_{t}=1-e^{-k_{1}t}$$

(5) $$F_{t}=k_{H}\sqrt{t}$$

(6) $$\sqrt[{3}]{F_{0}}-\sqrt[{3}]{F_{t}}=k_{HC}t$$

(7) $$F_{t}=k_{KP}t^{n}$$

where *F_t_* is the amount of 5-FU released at a specified time “*t*”, *F*_0_ is the initial amount of 5-FU in MIPs structure, *k*_0_, *k*_1_, *k*_H_, *k*_HC_, and *k*_KP_ are the release constants of corresponding equations, and *n* is the diffusion exponent.

### 2.6. Cytotoxicity Assays

The cytotoxicity assays were performed using the experimental procedure described in our previous research. Briefly, KB, HeLa, and MCF-7 cell lines were obtained from The European Collection of Cell Cultures (ECACC) supplied by Sigma-Aldrich (St. Louis, MO, USA). whereas A-549, U-87MG, and HDF cell lines were purchased from the American Type Cell Collection (ATCC) through LGC Standards. Approximately 0.1 mL of the diluted cell suspension (ca. 10,000 cells) was added to every well of the microtiter plate. A partial monolayer was formed after 24 h, and the supernatant was washed out. Then, 100 μL of 6 different 5-FU concentrations (0.1, 0.2, 1, 2, 10, and 20 μM) or number of MIPs that release the corresponding amount of 5-FU were added to the cells in microtiter plates. For NIPs blank experiments, the same mass of microparticles was used as for MIPs. Other experimental details were described in our previous work.

### 2.7. Statistical Analysis

One-way ANOVA with the post-hoc Tukey HSD test was used to test statistical significance. A *p*-value lower than 0.05 was considered as statistically significant.

## 3. Results and Discussion

The synthesized imprinted microparticles and corresponding non-imprinted microparticles present similar bands in the IR spectra (Figures S2 and S3), which indicates that the polymeric materials’ main structure for all samples is similar. For all synthesized materials, the stretching and bending O-H vibrations of carboxyl groups originating from MAA can be observed at 3562 and 1389 cm^−1^, respectively. These bands overlap with O-H stretching, and bending vibrations originated from hydroxyl groups of HEMA. The asymmetric stretching vibrations of CH_2_ groups can be observed at 2955 cm^−1^ for EGDMA-based microparticles and 2974 for TRIM-based microparticles. The stretching vibrations of C=O bonds are observed at 1730 cm^−1^ for EGDMA-based microparticles and 1734 cm^−1^ for TRIM-based microparticles. The symmetric and asymmetric C-O stretching vibrations of ester groups can be observed at 1258 and 1161 cm^−1^ for EGDMA-based microparticles, respectively. For TRIM-based microparticles, these signals are observed at 1268 and 1156 cm^−1^, respectively [46,47]. Drug-loaded MIP_EGDMA_ shows a band characteristic for 5-FU at 816 cm^−1^, whereas for drug-loaded MIP_TRIM_, these additional bands can be observed at 816, 553, and 471 cm^−1^.

**Figure 1:**
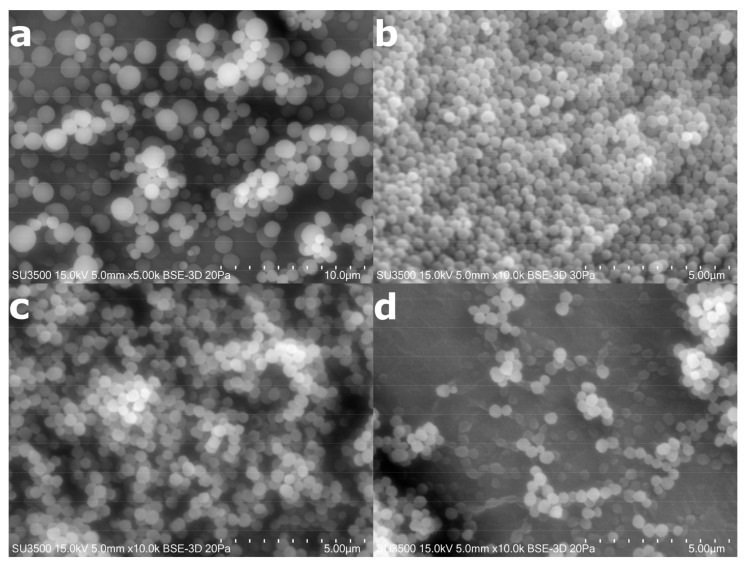
SEM images of (**a**) NIP_EGDMA_, (**b**) MIP_EGDMA_, (**c**) NIP_TRIM_, and (**d**) MIP_TRIM_.

SEM images of EGDMA- and TRIM-based microparticles are presented in Figure 1. For drug-loaded and drug-unloaded MIPs microparticles, no differences in SEM images were observed. SEM images of NIP_EGDMA_ present microparticles of 0.7–1.3 µm in diameter, whereas MIP_EGDMA_ microparticles are much smaller, ranging between 300 and 600 nm. This result clearly indicates that the presence of 5-FU in the polymerization mixture results in the formation of smaller microparticles. The NIP_TRIM_ microparticles are also smaller than those synthesized using EGDMA as a cross-linker, and their size ranges between 400 and 800 nm. Contrary to the previous observation, the presence of 5-FU in the polymerization mixture has not influenced the size of MIP_TRIM_ microparticles. This result may indicate that the presence of three methacrylate groups in the cross-linker structure that undergo polymerization makes the process less prone to the presence of additional substances in the reaction mixture that can affect the reaction.

The TG results obtained for EGDMA-based microparticles are presented in Figure S4, whereas those obtained for TRIM-based microparticles are shown in Figure S5. All EGDMA-based microparticles demonstrate one major decomposition step, from ca. 200 to ca. 450 °C. This step refers to the polymer structure’s decomposition and results in almost complete oxidation of organic material. For 5-FU-loaded MIP_EGDMA_ microparticles, an increased weight loss is observed in the initial decomposition step compared to drug-unloaded material. It probably results from the decomposition of 5-FU molecules. Compared to EGDMA-based microparticles, all TRIM-based microparticles are characterized by only one major decomposition step, that starts at around 300 °C and ends at around 475 °C, reflecting almost complete oxidation of the polymer material. For 5-FU-loaded MIP_TRIM_, only a slight increase in the weight loss of the initial decomposition step is observed compared with drug-unloaded material, which is connected with the decomposition of 5-FU molecules. The increase in the weight loss occurs within the 260–320 °C range, which is in accordance with the decomposition temperature of 5-FU reported in the literature [48].

### 3.1. Adsorption Isotherms

**Figure 2:**
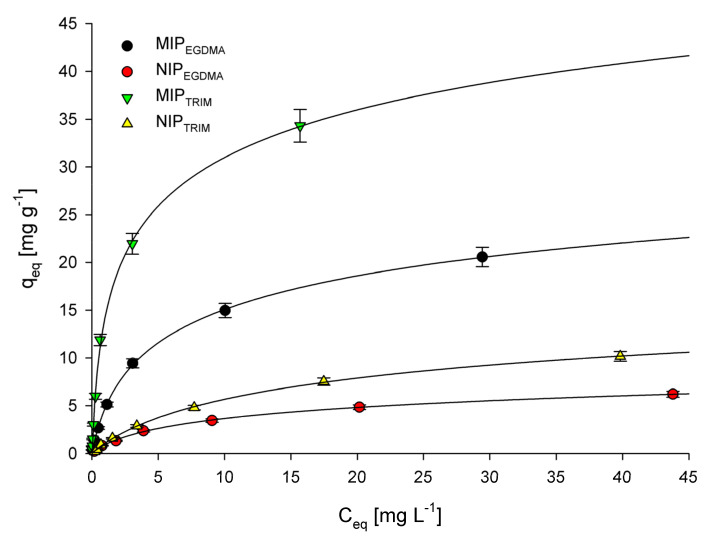
Adsorption isotherms of 5-FU onto MIP_EGDMA_, NIP_EGDMA_, MIP_TRIM_, and NIP_TRIM_.

The adsorption process’s characterization is achieved by plotting adsorption isotherms for experimental data obtained during adsorption of 5-FU by the microparticles at the equilibrium state. Figure 2 shows the relationship between the 5-FU equilibrium concentration and the amount of 5-FU adsorbed by 1 g of the appropriate adsorbent. To characterize the experimental data, Langmuir and Freundlich adsorption isotherm models were used for interpretation.

The following equation represents the Langmuir adsorption isotherm:

(8) $$\frac{C_{eq}}{q_{eq}}=\frac{C_{eq}}{q_{m}}+\frac{1}{Kq_{m}}$$

where *K* (L mg^−1^) is the binding equilibrium constant, *q_m_* (mg g^−1^) is the maximum amount of bonded 5-FU, *C_eq_* (mg L^−1^) is the equilibrium concentration of 5-FU, and *q_eq_* (mg g^−1^) is the amount of 5-FU adsorbed at the equilibrium concentration. Table 1 summarizes the values of *K*, *qm*, and R^2^ (correlation coefficients). The R^2^ values obtained for all synthesized microparticles are high (above 0.98), indicating that the Langmuir adsorption model nicely fits the experimental data. The confirmation of successful imprinting can be found in the high difference between the maximum adsorption capacity (*q_m_*) between MIPs and corresponding to them NIPs. For both MIPs–NIPs pairs, the *q_m_* value is around three times higher in favor of MIPs. There is also a significant difference in *q_m_* values between the corresponding materials synthesized using different cross-linkers. The calculated data clearly show that the maximum adsorption capacity values are higher for MIP_TRIM_ and NIP_TRIM_ than for MIP_EGDMA_ and NIP_EGDMA_, respectively. As a result, it can be concluded that a TRIM cross-linker allows constructing a polymer network that has a higher affinity to 5-FU and allows to imprint it more effectively than a network synthesized using the EGDMA cross-linker.

The following equations represent the Freundlich adsorption isotherm:

(9) $$q_{eq}=K_{f}C_{eq}^{1/n}$$

(10) $$\log q_{eq}=\log K_{f}+\frac{1}{n}\log C_{eq}$$

where *K*_f_ and *n* represent the Freundlich constants, *C_eq_* (mg L^−1^) is the equilibrium concentration of 5-FU, and *q_eq_* (mg g^−1^) is the amount of 5-FU adsorbed at the equilibrium concentration. Table 1 summarizes the values of *K*_f_, 1/*n*, and R^2^ (correlation coefficients). The R^2^ values obtained for MIP_EGDMA_, NIP_EGDMA_, and NIP_TRIM_ are higher than 0.97, suggesting that the experimental data can be fitted using the Freundlich adsorption model. On the other hand, the R^2^ values obtained for MIP_TRIM_ are lower than 0.94, indicating that the Freundlich adsorption model should not be used to characterize the experimental results. The 1/*n* value calculated from the Freundlich adsorption model is considered a measure of adsorption intensity or surface heterogeneity. Basically, the closer the 1/*n* value to zero, the more heterogeneous the surface [49]. Moreover, if the value of 1/*n* is higher than one, then adsorption is considered cooperative [50]. The 1/*n* values obtained for all microparticles are similar and are in the 0.60–0.73 range. This result indicates that all materials’ heterogeneity is similar and the adsorption of 5-FU on them follows a similar mechanism.

### 3.2. Adsorption Kinetics

Adsorption kinetics can be established by finding a relationship between the adsorption capacity of examined materials and the contact time with the adsorbate solution. For all synthesized microparticles, the plots of *q_t_* versus *t* were obtained and are shown in Figure S6. The experimental data were fitted using two adsorption kinetics models, the pseudo-first-order model given by Langergren and Svenska and the pseudo-second-order model based on the equilibrium adsorption. The following equation represents the pseudo-first-order model:

(11) $$\log\left(q_{e}-q_{t}\right)=\log q_{e}-\frac{k_{1}}{2.303}t$$

where *k*_1_ (h^−1^) is the pseudo-first-order rate constant, *q*_e_ (mg g^−1^) is the amount of 5-FU adsorbed at the equilibrium concentration, and q_t_ (mg g^−1^) is the amount of 5-FU adsorbed at time *t* (h). The *k*_1_ and R^2^ values are presented in Table 2. The calculated R^2^ values range between 0.979 and 0.993, which means that this model could be applied to characterize the kinetics of the adsorption of 5-FU on synthesized microparticles. However, after comparing these results with much higher R^2^ values calculated for the pseudo-second-order kinetic model, it becomes clear that the pseudo-first-order kinetic model only partially fits the experimental data obtained for all microparticles.

The following equation represents the pseudo-second-order kinetic model:

(12) $$\frac{t}{q_{t}}=\frac{1}{k_{2}q_{e}^{2}}+\frac{1}{q_{e}}t$$

where *k*_2_ (g mg^−1^ h^−1^) is the pseudo-second-order rate constant. The *k*_2_ and R^2^ values are presented in Table 2. The R^2^ values calculated using this model are much higher than those obtained for the pseudo-second-order kinetic model and are in the 0.994–0.999 range. This result indicates that the pseudo-second-order kinetic model should be used to characterize the adsorption of 5-FU by synthesized microparticles. For both MIPs and NIPs, the *k*_2_ values are quite similar and are within a level of 0.52–1.73, indicating that the kinetics of 5-FU adsorption for all materials is similar.

### 3.3. In Vitro Release Studies

**Figure 3:**
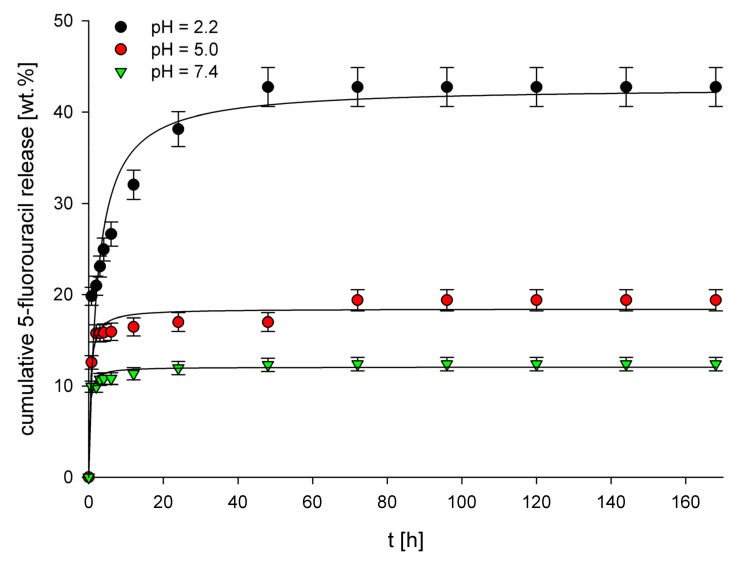
Release profiles of 5-FU from MIP_EGDMA_ at pH 2.2, 5.0, and 7.4.

**Figure 4:**
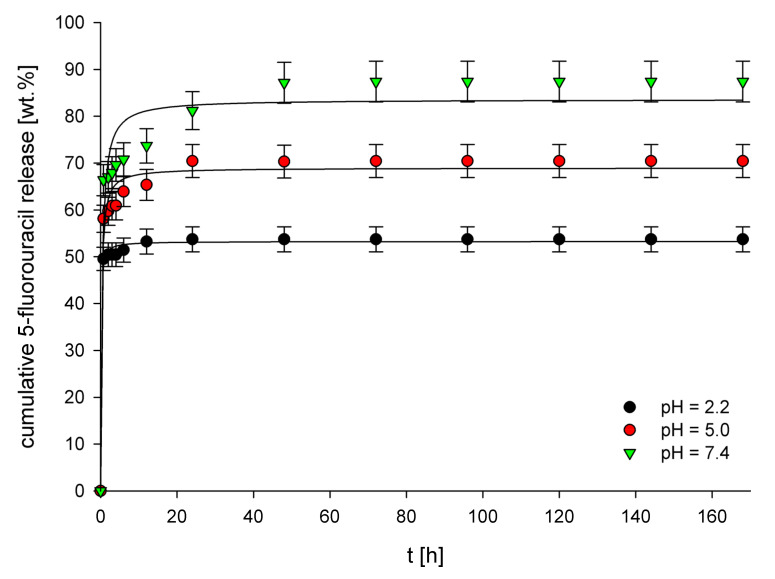
Release profiles of 5-FU from MIP_TRIM_ at pH 2.2, 5.0, and 7.4.

Three buffer solutions of pH 2.2 (simulated gastric fluid), pH 5.0 (simulated tumor interstitium of tumor cells) [51,52], and pH 7.4 (simulated intravenous conditions) were used as release media for in vitro release studies of 5-FU from the structures of drug-loaded imprinted materials. The release profiles of 5-FU from these materials (Figure 3 and Figure 4) clearly show entirely different properties of both MIPs depending on the cross-linker used. For each material and each examined pH value, a significant difference in the cumulative 5-FU release after statistical analysis has been noted (*p* < 0.05). For EGDMA-based MIPs (5-FU loading equal to 11.3 mg g^−1^), the highest cumulative release (ca. 40%) was observed for pH 2.2, whereas a much lower release, equal to 20% and 12%, was observed for pH 5.0 and 7.4, respectively. This result clearly shows that the higher the pH, the lower the cumulative release. The observed behavior is probably caused by the higher protonation of carboxylic groups present in the polymer structure at lower pH values. This process disrupts interactions between carboxylic groups and drug molecules, leading to increased drug release. The release profiles obtained for the examined pH values are different depending on the buffer pH. For pH 5.0 and 7.4, a very high initial burst release is observed, which means that after around 10 h, the release almost entirely reaches the final cumulative release values. On the other hand, the initial burst release at pH 2.2 is much lower, and after that, a steady release is observed, which lasts around 50 h, after which a final cumulative release value is reached. For TRIM-based MIPs (drug loading 24.2 mg g^−1^), a completely different behavior is observed regarding the dependence of pH of the release medium on the cumulative 5-FU release. The highest cumulative release (ca. 86%) was observed for pH 7.4, whereas for pH 5.0 and 2.2, these values were lower, equal to 70% and 53%, respectively. This behavior, almost opposite to the results obtained for MIP_EGDMA_, is presumably caused by the much higher hydrophobicity of the TRIM-based polymer network than the one observed for EGDMA-based polymers. As 5-FU is sparingly soluble in water, it possesses a relatively high affinity towards the hydrophilic environment, and therefore the hydrophobic TRIM-based polymer network does not form strong interactions with 5-FU drug molecules. As a result, at pH 7.4, the drug release from MIP_TRIM_ has the highest value because, at this pH value, the polymer network’s hydrophobicity is the highest. When the pH is lowered, the polymer’s structure becomes protonated, increasing its hydrophilicity, leading to a lower 5-FU release from its structure. Moreover, a rapid release at pH 2.2 can be observed, which is probably connected with breaking interactions occurring between MIPs cavities and 5-FU. As a result, during release, the amount of 5-FU maintained in the MIP_TRIM_ structure is physisorbed primarily on its structure due to the polymer’s increased hydrophilicity. The higher hydrophobicity of the TRIM-based polymer network than the EGDMA-based network also explains the much higher cumulative percentage release of 5-FU for MIP_TRIM_ than the one observed for MIP_EGDMA_.

Several mathematical models were used to fit the experimental data obtained from the 5-FU release from MIP_EGDMA_ and MIP_TRIM_. The release profiles were fitted with zero-order, first-order, Higuchi, Hixson–Crowell, and Korsmeyer–Peppas release models. The values of corresponding release constants (*k*), correlation coefficients (R^2^), and the diffusion exponents (*n*) are presented in Table 3. For almost all pH values and both imprinted materials, the highest R^2^ values were calculated for the Korsmeyer–Peppas release model, which indicates that the experimental data is best fitted with this model. The only exception is the release of 5-FU from MIP_TRIM_ at pH 5.0, for which a higher R^2^ value was obtained for the Higuchi model (R^2^ = 0.977) compared to the Korsmeyer–Peppas model (R^2^ = 0.956). However, the differences in the R^2^ values are not significant; therefore, it can be concluded that the experimental data is nicely fitted with both of these models. These results indicate that the release mechanism of 5-FU from both MIPs at all examined pH values is similar. Moreover, for all MIPs at all examined pH values, the diffusion exponent value in the Korsmeyer–Peppas model was lower than 0.45, indicating a Fickian diffusion-controlled release mechanism.

### 3.4. In Vitro Cell Viability

Cytotoxicity against U87 MG, HeLa, KB, A-549, and MCF-7 cancer cell lines, and HDF normal cell lines, was examined for drug-loaded and drug-unloaded MIPs and pure 5-FU. Table 4 summarizes the IC_50_ values. Both drug-loaded MIPs present very high cytotoxicity against cancer cell lines, contrary to the result obtained for drug-unloaded MIPs. The results clearly show that drug-loaded MIP_TRIM_ shows almost identical cytotoxicity against HeLa cancer cell lines as pure 5-FU. In contrast, its cytotoxicity against normal HDF cell lines is nearly three times lower than that measured for 5-FU. As a result, it can be concluded that MIP_TRIM_ can be a good candidate as a DDS for 5-FU because it allows maintaining high cytotoxicity against HeLa cell lines while lowering the cytotoxicity against healthy cells.

## 5. Conclusions

We have shown that microparticles imprinted with 5-FU can be applied for the prolonged release of this drug. The microparticles synthesized using the TRIM cross-linker showed higher adsorption properties towards 5-FU than those synthesized using EGDMA. The experiments proved that prolonged drug release could last up to 50 h when an EGDMA cross-linker is used. The calculated highest cumulative release was highly dependent on the cross-linker applied during the synthesis. For EGDMA-based MIPs, the highest cumulative release was observed at pH 2.2, lower at pH 5.0, and the lowest at pH 7.4. Opposite results were obtained for TRIM-based MIPs, as the highest cumulative release was obtained for pH 7.4 and the lowest for pH 2.2. Moreover, the overall cumulative release was much higher for TRIM-based than EGDMA-based MIPs. The 5-FU release from all examined materials at all pH values was well fitted with the Korsmeyer–Peppas model. The IC_50_ values proved that drug-loaded MIP_TRIM_ possesses high cytotoxic activity against cancer cell lines, while lowering the toxicity towards normal HDF cell lines. Therefore, it has been proven that the selection of a cross-linker has a significant impact on the final properties of microparticles and has to be considered during the design of materials used for drug delivery.

## Figures and Tables

**Table 1:** Parameters of 5-FU adsorption by MIPs and NIPs.

Polymer	Langmuir			Freundlich		
	q_m_ (mg g^−1^)	K (L mg^−1^)	R^2^	K_f_ (mg g^−1^ (L mg^−1^)^1/n^)	1/n	R^2^
MIP_EGDMA_	22.57 ± 1.96	0.285 ± 0.025	0.991	3.59 ± 0.32	0.62 ± 0.06	0.973
NIP_EGDMA_	7.00 ± 0.64	0.142 ± 0.013	0.989	0.82 ± 0.08	0.60 ± 0.06	0.977
MIP_TRIM_	37.30 ± 3.46	0.676 ± 0.064	0.997	9.72 ± 0.92	0.66 ± 0.06	0.937
NIP_TRIM_	12.89 ± 1.63	0.088 ± 0.008	0.992	0.95 ± 0.09	0.73 ± 0.07	0.972

**Table 2:** Kinetic parameters calculated for pseudo-first-order and pseudo-second-order models.

Polymer	Pseudo-First-Order Kinetic Model		Pseudo-Second-Order Kinetic Model	
	k_1_ (h^−1^)	R^2^	k_2_ (g mg^−1^ h^−1^)	R^2^
MIP_EGDMA_	3.46 ± 0.31	0.987	1.03 ± 0.09	0.998
NIP_EGDMA_	1.50 ± 0.14	0.979	1.00 ± 0.09	0.997
MIP_TRIM_	2.47 ± 0.22	0.993	0.52 ± 0.05	0.999
NIP_TRIM_	1.74 ± 0.15	0.980	1.73 ± 0.14	0.994

**Table 3:** Release kinetic data of 5-FU from MIP_EGDMA_ and MIP_TRIM_.

Polymer	pH	Zero-Order		First-Order		Higuchi		Hixson–Crowell		Korsmeyer–Peppas		
		k_0_ (h^−1^)	R^2^	k_1_ (h^−1^)	R^2^	k_H_ (h^−1/2^)	R^2^	k_HC_ (h^−1/3^)	R^2^	k_KP_ (h^−n^)	n	R^2^
MIP_EGDMA_	2.2	0.106 ± 0.093	0.621	1.63 × 10^−3^ ± 0.14 × 10^−3^	0.634	1.73 ± 0.15	0.791	2.19 × 10^−3^ ± 0.20 × 10^−3^	0.630	20.7 ± 1.8	0.16 ± 0.2	0.900
	5.0	0.110 ± 0.009	0.405	1.30 × 10^−3^ ± 0.11 × 10^−3^	0.413	0.77 ± 0.07	0.555	1.91 × 10^−3^ ± 0.17 × 10^−3^	0.410	13.9 ± 1.2	0.07 ± 0.01	0.726
	7.4	0.047 ± 0.004	0.784	0.53 × 10^−3^ ± 0.05 × 10^−3^	0.787	0.40 ± 0.04	0.910	0.79 × 10^−3^ ± 0.07 × 10^−3^	0.786	9.9 ± 0.9	0.06 ± 0.01	0.944
MIP_TRIM_	2.2	0.085 ± 0.008	0.670	1.77 × 10^−3^ ± 0.14 × 10^−3^	0.674	0.70 ± 0.07	0.803	1.93 × 10^−3^ ± 0.16 × 10^−3^	0.661	49.3 ± 4.7	0.03 ± 0.01	0.896
	5.0	0.494 ± 0.42	0.944	6.82 × 10^−3^ ± 0.61 × 10^−3^	0.783	3.01 ± 0.28	0.977	1.46 × 10^−2^ ± 0.11 × 10^−2^	0.966	56.5 ± 5.2	0.06 ± 0.01	0.956
	7.4	0.130 ± 0.011	0.705	6.40 × 10^−3^ ± 0.59 × 10^−3^	0.737	1.96 ± 0.16	0.867	5.78 × 10^−3^ ± 0.53 × 10^−3^	0.728	64.8 ± 6.2	0.06 ± 0.01	0.947

**Table 4:** The IC_50_ values (µg mL^−1^) obtained for drug-loaded and unloaded MIPs and pure 5-FU. Standard deviations are presented in brackets.

Material	U87 MG	HeLa	KB	A-549	MCF-7	HDF
unloaded MIP_EGDMA_	62.13 (0.11)	67.02 (0.49)	67.39 (0.07)	71.08 (0.73)	63.35 (0.19)	98.19 (0.51)
unloaded MIP_TRIM_	81.12 (0.04)	69.02 (0.31)	81.01 (1.04)	77.82 (0.59)	81.93 (0.94)	102.02 (0.87)
loaded MIP_EGDMA_	0.38 (0.07)	1.02 (0.11)	0.41 (0.16)	0.31 (0.91)	0.19 (0.01)	0.44 (0.49)
loaded MIP_TRIM_	0.50 (0.04)	0.16 (0.01)	0.21 (0.17)	0.26 (0.19)	0.22 (0.01)	1.94 (0.55)
5-FU	0.07 (0.01)	0.16 (0.13)	0.03 (0.01)	0.08 (0.03)	0.08 (0.07)	0.71 (0.05)

## Data Availability

The additional data that support the findings of this study are available from the corresponding author upon request.

## Supplementary Materials

The following supporting information can be downloaded at: https://www.mdpi.com/article/10.3390/polym14051027/s1, Figure S1: Chemical structures of 5- fluorouracil, functional monomers, and cross-linkers. Figure S2: FT-IR spectra of loaded MIP_EGDMA_, unloaded MIP_EGDMA_, and NIP_EGDMA_. Figure S3: FT-IR spectra of loaded MIP_TRIM_, unloaded MIP_TRIM_, and NIP_TRIM_. Figure S4: Results of thermogravimetric analysis for NIP_EGDMA_, unloaded MIP_EGDMA_, and loaded MIP_EGDMA_. Figure S5: Results of thermogravimetric analysis for NIP_TRIM_, unloaded MIP_TRIM_, and loaded MIP_TRIM_. Figure S6: The relationship between q_t_ and t obtained for MIP_EGDMA_, NIP_EGDMA_, MIP_TRIM_, and NIP_TRIM_ during adsorption kinetics experiments.
