# Supplementary material for: EGDMA- and TRIM-Based Microparticles Imprinted with 5-Fluorouracil for Prolonged Drug Delivery
Source: Polymers (Basel). 2022 Mar 4;14(5):1027. doi: 10.3390/polym14051027 (PMC8914908; doi:10.3390/polym14051027)
Supplement: Supplementary file 1 [file polymers-14-01027-s001.zip › polymers-1560976-supplementary.pdf]

polymers

Supplementary

EGDMA- and TRIM-Based Microparticles Imprinted with 5-Fluorouracil for Prolonged Drug Delivery

Michał Cegłowski [1,*], Joanna Kurczewska [1], Aleksandra Lusina [1], Tomasz Nazim [1] and Piotr Ruszkowski [2]

[1] Faculty of Chemistry, Adam Mickiewicz University, 61-614 Poznan, Poland; asiaw@amu.edu.pl (J.K.); aleksandra.lusina@amu.edu.pl (A.L.); tomasz.nazim@amu.edu.pl (T.N.)
[2] Department of Pharmacology, Poznan University of Medical Sciences, 61-614 Poznan, Poland; pruszkowski@gmail.com
* Correspondence: michal.ceglowski@amu.edu.pl; Tel.: +48-61-8291-799

Figures

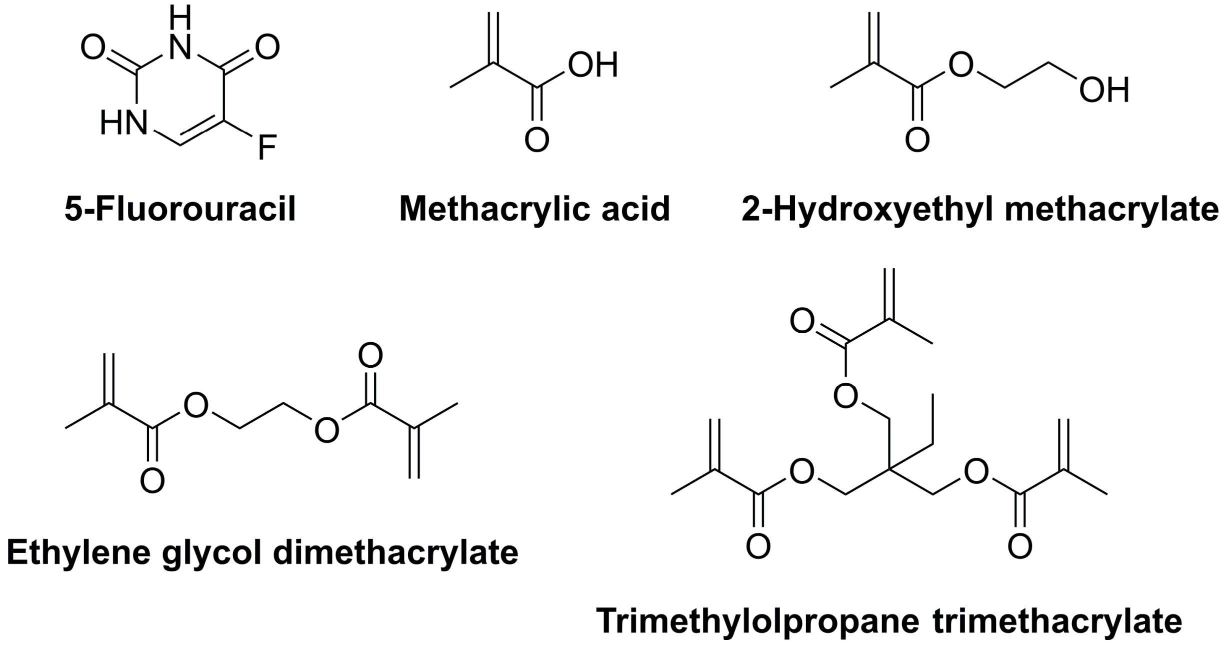

Figure S1. Chemical structures of 5- fluorouracil, functional monomers and cross-linkers.

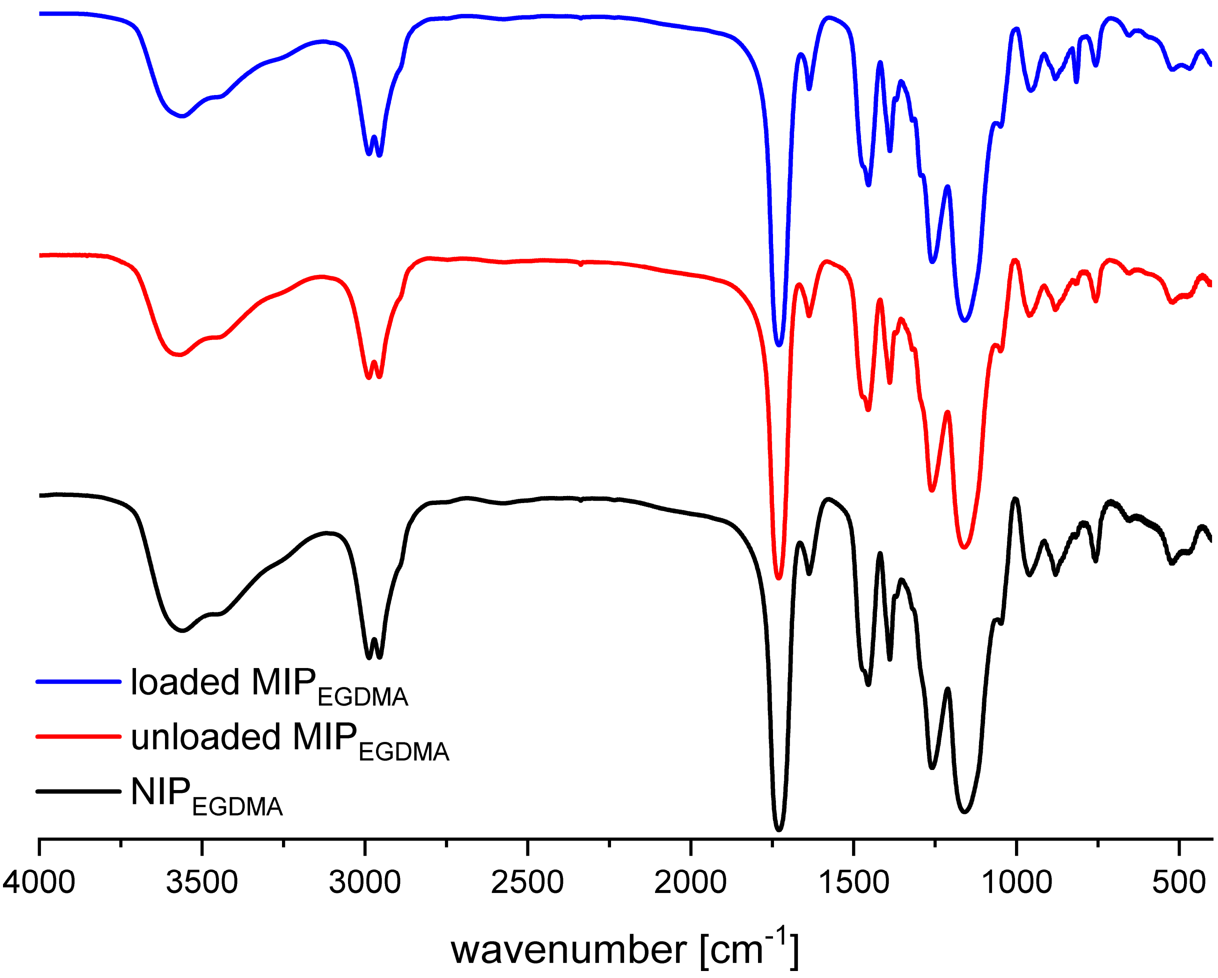

Figure S2. FT-IR spectra of loaded MIP_{EGDMA}, unloaded MIP_{EGDMA}, and NIP_{EGDMA}.

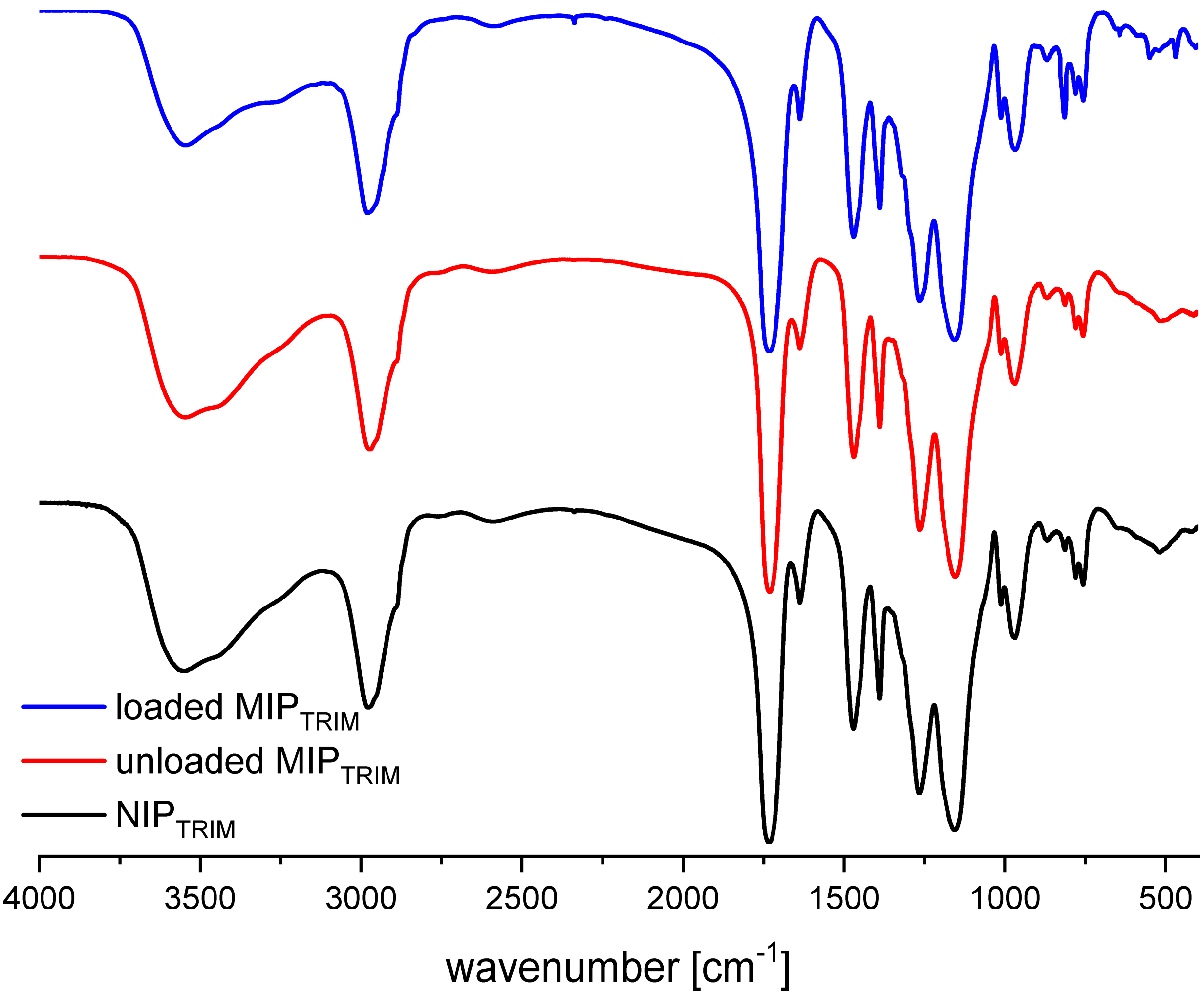

Figure S3. FT-IR spectra of loaded MIP_{TRIM}, unloaded MIP_{TRIM}, and NIP_{TRIM}.

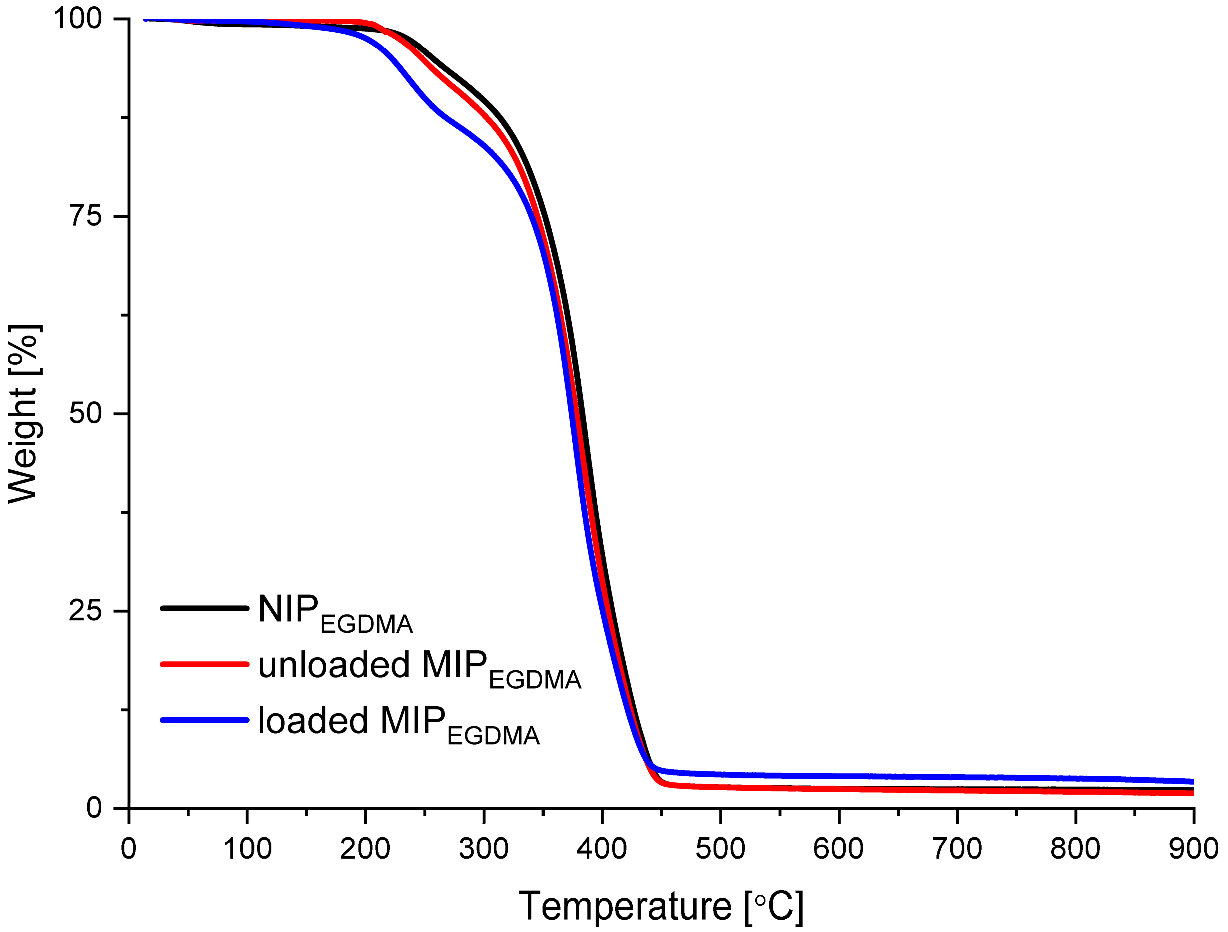

Figure S4. Results of thermogravimetric analysis for NIP$_{EGDMA}$, unloaded MIP$_{EGDMA}$, and loaded MIP$_{EGDMA}$.

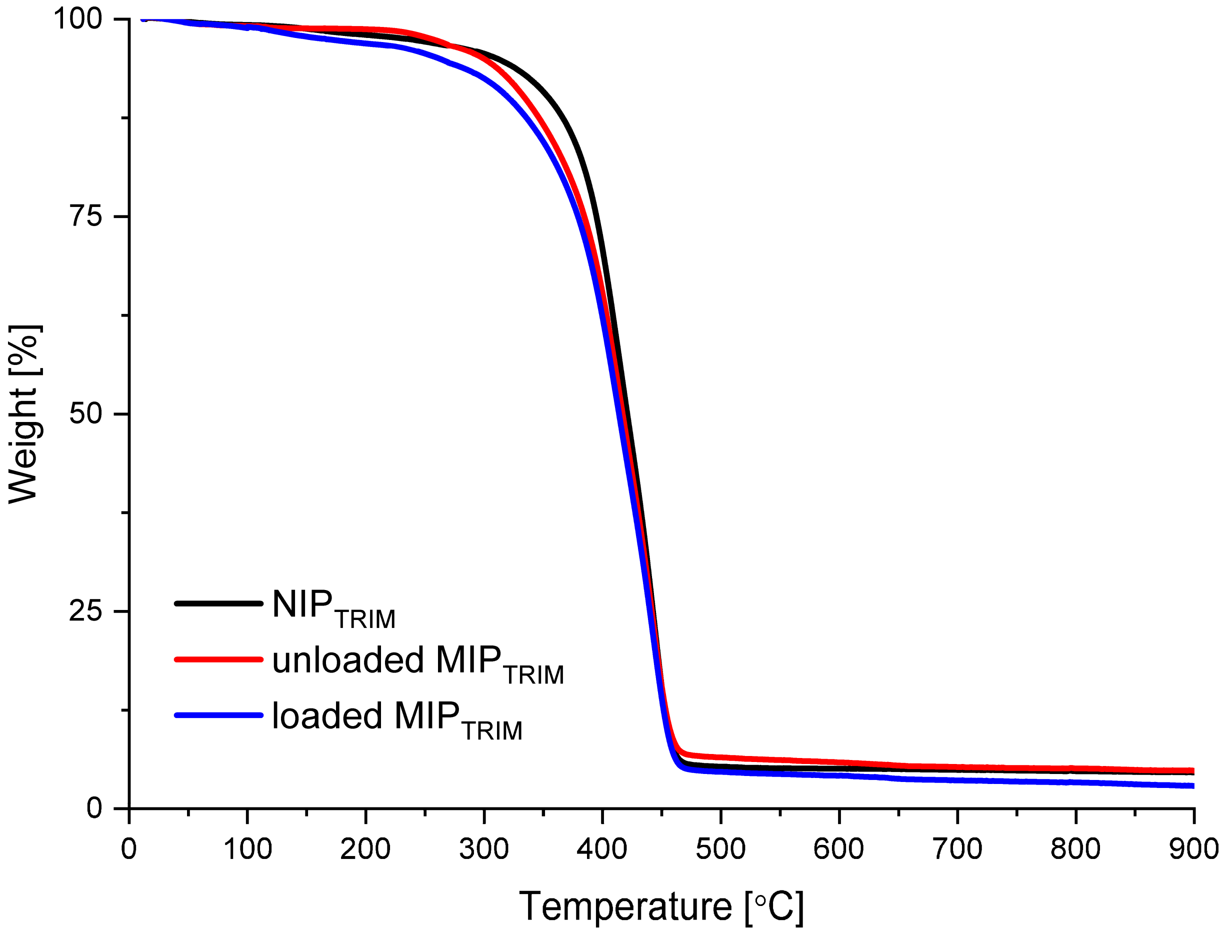

Figure S5. Results of thermogravimetric analysis for NIP$_{TRIM}$, unloaded MIP$_{TRIM}$, and loaded MIP$_{TRIM}$.

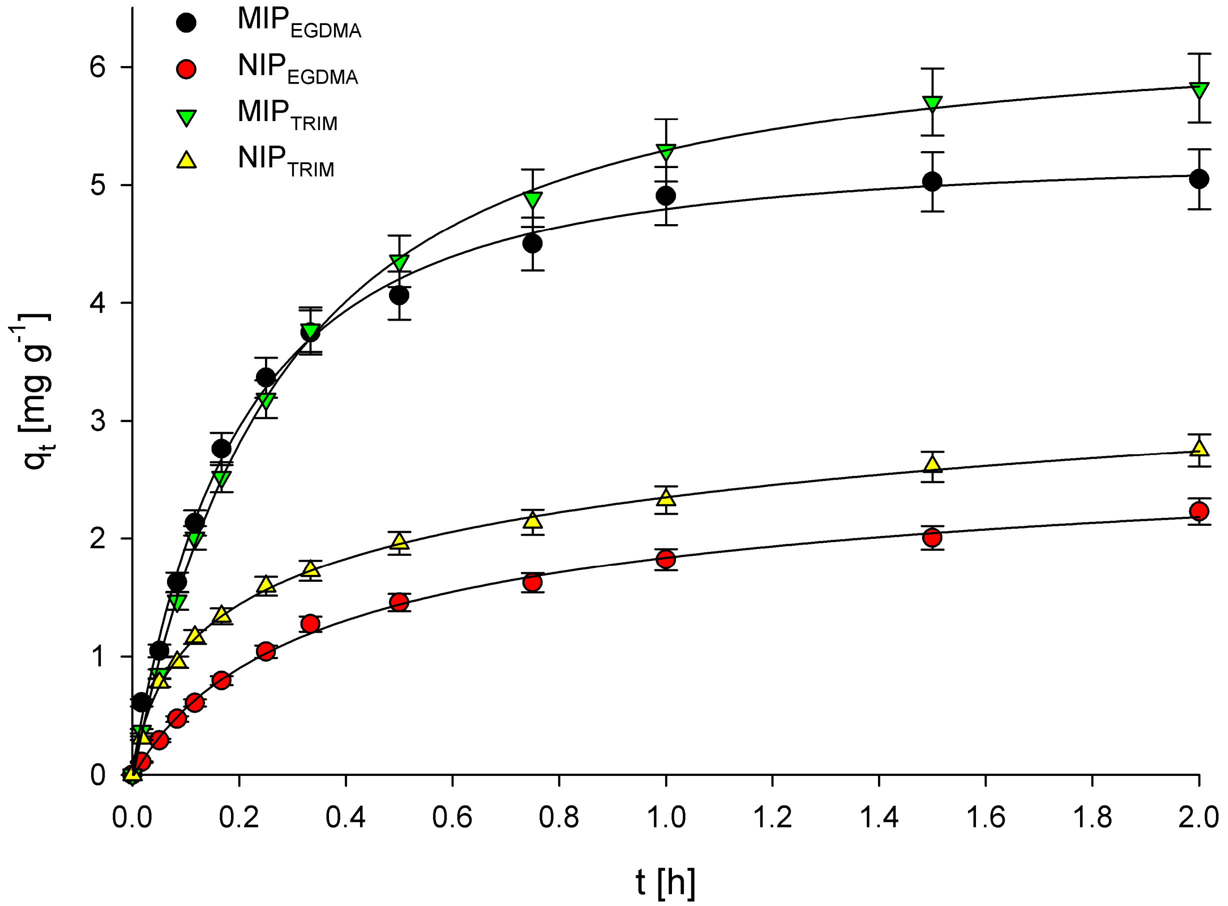

Figure S6. The relationship between q_t and t obtained for MIP_{EGDMA}, NIP_{EGDMA}, MIP_{TRIM}, and NIP_{TRIM} during adsorption kinetics experiments.
